# COVID-19 Vaccine Hesitancy and Vaccination Coverage in India: An Exploratory Analysis

**DOI:** 10.3390/vaccines10050739

**Published:** 2022-05-09

**Authors:** Pritu Dhalaria, Himanshu Arora, Ajeet Kumar Singh, Mansi Mathur, Ajai Kumar S.

**Affiliations:** Immunization Technical Support Unit, Ministry of Health & Family Welfare, Government of India, New Delhi 110070, India; pritu_dhalaria@in.jsi.com (P.D.); himanshu_arora@in.jsi.com (H.A.); mansi_mathur@in.jsi.com (M.M.); ajai_kutty@in.jsi.com (A.K.S.)

**Keywords:** vaccine, hesitancy, coverage, poverty, health, decision

## Abstract

Our paper examines the key determinants of COVID-19 vaccination coverage in India and presents an analytical framework to probe whether vaccine hesitancy, socioeconomic factors and multi-dimensional deprivations (MPI) play a role in determining COVID-19 vaccination uptake. Our exploratory analysis reveals that COVID-19 vaccine hesitancy has a negative and statistically significant impact on COVID-19 vaccination coverage. A percentage increase in vaccine hesitancy can lead to a decline in vaccination coverage by 30 percent. Similarly, an increase in the proportion of people living in multi-dimensional poverty reduces the COVID-19 vaccination coverage. A unit increase in MPI or proportion of people living in acute poverty leads to a mean decline in vaccination coverage by 50 percent. It implies that an increase in socioeconomic deprivation negatively impacts health outcomes, including vaccination coverage. We additionally demonstrated that gender plays a significant role in determining how access to digital technologies such as the internet impacts vaccine coverage and hesitancy. We found that, as males’ access to the internet increases, vaccination coverage also increases. This may be attributed to India’s reliance on digital tools (COWIN, AAROGYA SETU, Imphal, India) to allocate and register for COVID-19 vaccines and the associated digital divide (males have greater digital excess than females). Conversely, females’ access to the internet is statistically significant and inversely associated with coverage. This can be attributed to higher vaccine hesitancy among the female population and lower utilization of health services by females.

## 1. Introduction

The coronavirus (SARS-CoV-2) manifested in the Hubei province (Wuhan) of China sometime in October 2019. In a short span of three months, the virus had spread to over 100 countries with more than 2 lakh cases reported globally, resulting in the World Health Organization (WHO) declaring it a pandemic. The scale of transmission, infections and mortality overwhelmed the healthcare systems, leading to countries enforcing complete lockdowns to stop the spread of the coronavirus. In the absence of vaccines, the strategy followed by governments all over the world to reduce the risk of contagion was non-pharmaceutical interventions (NPIs), such as enforcing restrictions on movements, the use of facial masks, mandatory quarantines, physical distancing, travel restrictions and lockdowns. The NPIs were able to somewhat slow down the progression of the coronavirus, but at a huge economic cost [1]. In the meantime, scientific and medical institutions were engaged in rapidly developing an effective vaccine against the coronavirus [2]. By March 2021, multiple countries including the UK, USA, India, Russia and China have started to develop vaccines. 

India is among the few countries to develop its own indigenous COVID-19 vaccines—Covishield (Serum Institute, Pune, India) and Covaxin (Bharat Biotech, Hyderabad, India)—and started to vaccinate its people against coronavirus beginning in January 2021. In the first phase of the immunization plan, vaccines were administered to the frontline and healthcare workers, followed by the elderly and people with co-morbidities. Subsequently, the vaccination was allowed for all other categories and age groups. With an adult population of 940 million, India made remarkable progress by achieving 100% first dose coverage and 80% full vaccination coverage, administering 1.7 billion doses of total COVID-19 vaccines to over 940 million individuals (as of 9 February 2022). However, India has been struggling with demand-side barriers such as vaccine hesitancy and widespread misinformation surrounding COVID-19 vaccines. Official data from the Ministry of Health and Family Welfare (MOHFW) suggest interstate variations in vaccination coverage, with some states such as Nagaland, Manipur, Meghalaya, Mizoram, Jharkhand, Uttar Pradesh, Bihar, Punjab, etc. lagging.

Hesitancy toward the newly developed COVID-19 vaccines is a global phenomenon. Available literature suggests that vaccination hesitancy varies significantly across the countries: United States (21%), United Kingdom (25%), Russia (45%), Poland (44%), France (41%), Kuwait (76%) and, Jordan (71%) [3]. The WHO defined vaccine hesitancy as a “delay in acceptance or refusal of vaccination despite the availability of vaccination services.” A review by Solis Arce et al. investigated the questions pertaining to COVID-19 vaccine hesitancy, such as whether people are willing to be vaccinated, the reasons why they are willing or unwilling to do so, and the most trusted sources of information in their decision-making, using a common set of survey items deployed between June 2020 and January 2021, across 13 studies carried out in Africa, South Asia, Latin America, Russia, and the United States. Overall, they found that the average acceptance rate across the full set of studies in low- and middle-income countries (LMICs) was 80.3%, with the lowest acceptance rates in Burkina Faso (66.5%) and Pakistan (66.5%); moreover, the acceptance rate in India (84%) was higher than that of samples from the United States (64.6%) and Russia (30.4%) [4]. The result also shows that vaccine acceptance is explained mainly by an interest in personal protection against COVID-19, whereas concerns about side effects are the most common reasons for hesitancy, and health workers are the most trusted sources of guidance about vaccines against COVID-19.

In India too, a significant proportion of the eligible population is hesitant toward COVID-19 vaccines. For instance, a survey conducted by local circles in India in October 2021 estimated over 75 million eligible candidates to be vaccine-hesitant. According to the survey, the candidates who refused to take the COVID-19 vaccine cited rumours, the ineffectiveness of vaccines against the new COVID-19 variants (16%), death and infertility following vaccination (23%), and vaccines being unsafe for people with co-morbidities (23%) [5] as deterrents. Another study conducted on 1638 adults to assess the extent of COVID-19 vaccine hesitancy in India indicated that 37% of the respondents were either not sure or refused to get the COVID-19 vaccines, translating into roughly more than 200 million adults (using power analysis-95% confidence level, alpha = 0.025, power = 0.80) across the country. In total, 71% of the study participants reported at least one concern regarding vaccines, with the most common concerns being safety, side effects, and effectiveness. The study further reported that vaccine hesitancy is influenced by socioeconomic factors such as poverty, gender, geography, employment, and education status. Vaccine hesitancy was statistically significantly higher for females (38%), urban dwellers (40%), salaried (41%), graduate and above (41%) [6]. 

In addition to socioeconomic factors, available literature suggests that the availability of digital tools such as the internet and smartphones helps in enhancing awareness and access to vaccines. However, it also manifests challenges, mainly rumours, misinformation, anti-vax sentiments, fake news, and incomplete information regarding vaccines [7]. In fact, the COVID-19 pandemic is the first pandemic in history in which digital technologies (internet, smartphones, etc.) and social media are being used enormously to keep people informed and connected. However, the same digital technologies are amplifying ‘infodemic-deliberate attempts to disseminate misinformation to undermine the pandemic response.’ Evidence suggests that misinformed narratives have negative consequences on health outcomes and promote undesirable behaviour, including vaccine hesitancy. 

Given India’s past experience with regard to adult and childhood vaccination, where a sizeable proportion of the population has displayed strong hesitancy, the likelihood of a significant population believing in falsehoods, rumours, and fake narratives regarding COVID-19 vaccines is strong. A significant proportion of the eligible population displayed anti-COVID-19 vaccine sentiments fueled by misinformation, rumours and fake news spread over the internet. For instance, the indigenous COVID-19 vaccines suffered from the widespread misinformation that it contains pork gelatin and cow serum, both considered to be forbidden by religious law and hurt religious sentiments [8]. Another study, approved by the All-India Institute of Medical Science, Delhi, and conducted on 1294 individuals to assess the knowledge and attitudes regarding COVID-19 vaccines, found that the decision to get vaccinated is influenced by information available on social media platforms and What’s App (74%). The influence of digital tools such as social media was significantly associated (*p* < 0.001) with the place of residence, age, and socioeconomic status [9]. 

The available literature cited above suggests that COVID-19 vaccination coverage varies considerably according to geography, socioeconomic conditions, gender, class, rural-urban divide, the extent of vaccine hesitancy, and access to digital tools such as the internet or social media [10,11]. However, there is a dearth of studies in India that can establish causal impacts between COVID-19 vaccine coverage and covariates such as vaccine hesitancy, geography, education attainment, socioeconomic status, and access to digital tools. The majority of the studies report the findings of the surveys without making causal inferences. Identifying the factors that interplay and result in high hesitancy rates among a population can allow the formulation of a directed intervention to increase vaccination uptake rates. It is also essential in planning strategies to increase vaccine acceptability in terms of coverage across populations belonging to different socioeconomic groups. 

It is against this backdrop that our paper aims to fill the void and contributes to the existing literature in multiple ways. First, we use econometric models with varying assumptions to explore the factors determining COVID-19 vaccine coverage in India and estimate the causal relationship between them. Second, we estimate the impact of social and economic variables on COVID-19 vaccination coverage in Indian states. Our analysis uses a demand and supply-side framework to probe whether vaccine hesitancy, access to the internet and multi-dimensional deprivations play any statistically significant role in determining COVID-19 vaccination uptake or coverage.

## 2. Method and Materials 

The present analysis is based on the real-time dataset of the number of people vaccinated against COVID-19 infection, reported for Indian states on the Cowin dashboard and the Ministry of Health and Family Welfare, India. The extent of COVID-19 vaccine hesitancy or the proportion of the population hesitant to accept COVID-19 vaccines is estimated from the COVID-19 Consumption Survey (CSS). The CSS is a cross-section survey conducted daily by academic institutions—Carnegie Mellon University, Delphi Research Centre, and University of Maryland’s Joint Program in Survey methodology, with Facebook providing its platform to recruit participants. Each day, randomly selected users get an invitation to participate in the survey at the top of their Facebook News Feed. The survey questionnaire is standardised and asks various questions related to health symptoms, COVID-19 symptoms, testing, COVID-19 vaccines, and vaccine behaviour. The same is developed by public health and survey experts and was reviewed and approved by the Institutional Review Boards of both the University of Maryland and Carnegie Mellon University [12]. We have used the CSS survey findings (as secondary data) to explore the national and sub-national trends in vaccine hesitancy due to its reliable sampling framework. It leverages Facebook’s active base of over 320 million users in India as the sampling frame. To provide adequate geographic coverage, stratified random sampling within Indian states is used. To account for demographic differences between the sampling frame of Facebook users (i.e., the Facebook Active User Base (FAUB) aged 18+) and India’s population, and for bias related to non-response and coverage, the survey employs a two-stage weighting process. In the first stage, inverse propensity score weighting is used to adjust for non-response bias by making the sample more representative of the FAUB. The covariates used in this step are obtained from internal Facebook data, including self-reported age, gender, geographical variables, and other Facebook user characteristics that have been found to correlate with survey responses in the past. In the second stage, post-stratification is used to balance the state-level distribution of age and gender among the Facebook population based on the UN population projections (for India), 2020 [13]. The resulting weights are provided as part of the microdata and are used to adjust estimates so that the survey population is representative of the general population thereby adjusting both for the differences between the general population and Facebook users and for the propensity of a Facebook user to take the survey in the first place. We understand that CSS design is subject to limitations. We understand these limitations and therefore used the data only for the following purposes in the study: To track trends over time; increase or decrease in reported vaccination attitudes, changes in the reported level of vaccine hesitancy in the Indian states; Make a comparison across geographies (Indian states) to identify regions with higher or lower values of hesitancy [13].

In a systematic review on validation of web-based surveys, the study highlighting the advantages of the CSS, demonstrated how large online surveys provide continuous, real-time indicators of important outcomes that are not subject to public health reporting delays and backlogs. The CSS offers high value as a supplement to official reporting data by supplying essential information about behaviour, attitude towards policy and preventive measures, economic impacts, and key indicators otherwise not reported in public health surveillance systems [14]. 

We assessed the CSS findings from January 2020 to December 2021 to estimate the prevalence of vaccine hesitancy in India. The survey maps the vaccine hesitancy by asking respondents “If a vaccine to prevent COVID-19 were offered to you, would you choose to get vaccinated. The respondents were asked to respond with the following options: Yes, definitely; Yes, probably; No, probably not; No, definitely not. Vaccine hesitancy is estimated based on probably not and definitely not responses. Data were aggregated by month to evaluate time trends in COVID-19 vaccine uptake and intent. Data were treated as repeat cross-sectional surveys and estimates were generated using survey weight.

The data on socioeconomic deprivation i.e., multi-dimensional poverty (MPI) headcount is from the NITI AAYOG report [15]. The survey data from the National Family Health Survey (NFHS) round 5 has been used to capture the state-level estimates on routine immunisation, male and female years of schooling, and male and female access to the internet. 

### The Model

First, we deployed the ordinary least square regression model to explore the factors determining COVID-19 vaccine coverage in Indian states, using actual vaccination data available on the Cowin dashboard on daily basis. We have used linear and log regression models separately to explore the causal relationship between the proportion of the population fully vaccinated against COVID-19 and the extent of vaccine hesitancy; the proportion of the population categorised as multi-dimensionally poor; vaccine wastage rate; access to the internet. 

A. Dependent Variable

The dependent variable for our analysis is the COVID-19 vaccination coverage i.e., the proportion of the population fully vaccinated against COVID-19 infection. 

B. Independent variable. 

Our analysis uses demand and supply-side factors that can impact COVID-19 vaccination coverage. To capture the demand side, we have used the extent of vaccine hesitancy i.e., the proportion of the population hesitant to accept COVID-19 vaccines from the COVID-19 consumption survey. Higher COVID-19 vaccine hesitancy is associated with lower vaccination coverage. We have also used several socioeconomic variables as predictors of COVID-19 vaccine coverage. To capture the supply side, we have used the multi-dimensional poverty index of the NITI Aayog. MPI indexed the socioeconomic deprivation in Indian states using the following 3 dimensions: health, education and standard of living by assigning equal weights. These dimensions are further subdivided into the following 12 core indicators: undernutrition, child mortality, years of schooling, access to cooking fuel, sanitation, drinking water, electricity, housing, asset, financial inclusion, etc. [15]. Intuitively, states with higher MPI scores or a higher proportion of people living in multi-dimensional poverty tend to perform poorly on healthcare including immunisation. Other socioeconomic indicators such as years of schooling and access to the internet are also used as independent variables to capture the impact of literacy and technology on the COVID-19 vaccination coverage. Both the variables have the potential to explain the spatial differences in vaccination coverage. For instance, access to the internet can increase the likelihood of getting vaccinated as it helps in timely registration for vaccines, accessing vaccine certificates, etc. However, internet access can also reduce the likelihood of getting vaccinated as people can get influenced by fake news and rampant misinformation on COVID-19 vaccines. If the former dominates the latter, vaccination coverage may increase and vice versa. Education characteristics may likewise impact awareness regarding healthcare and COVID-19 infection which can further influence decision-making around vaccination.

(1) $$Y_{i}=\beta_{0}+\beta_{1}x_{1}+\beta_{2}x_{2}+\beta_{3}x_{3}+\beta_{4}x_{4}+\beta_{5}x_{5}+\varepsilon_{i}$$

(2) $$ln\;Y_{i}=\beta_{0}+\left(ln\right)\;\beta_{1}x_{1}+\left(ln\right)\;\beta_{2}x_{2}+\left(ln\right)\;\beta_{3}x_{3}+\left(ln\right)\;\beta_{4}x_{4}+\left(ln\right)\;\beta_{5}x_{5}+\varepsilon_{i}$$

where,

*Y_i_* = Proportion of the Population Fully Vaccinated.

*x*_1_ = Vaccine Hesitancy; $\beta_{1}$ = slope coefficient that represents a change in output associated with a change in input unit.

*x*_2_ = Multi-dimensional Deprivation Index Score; $\beta_{2}$ = slope coefficient that represents a change in output associated with a change in input unit.

*x*_3_ = Female Access to the Internet; $\beta_{3}$ = slope coefficient that represents a change in output associated with a change in input unit.

*x*_4_ = Male Access to the Internet; $\beta_{4}$ = slope coefficient that represents a change in output associated with a change in input unit.

*x*_5_ = Vaccine wastage rate; $\beta_{5}$ = slope coefficient that represents a change in output associated with a change in input unit.

$\varepsilon_{i}=error\;term$; ln is the natural log.

Next, using the loess regression model, we explored the components that may have an individual impact on COVID-19 vaccine hesitancy. We have used determinants such as literacy, access to the internet, and multi-dimensional poverty index as individual regressors. Our objective in this model is to represent the relationship between the response variable [Vaccine Hesitancy] and the one or more predictor variables [Schooling, Internet, etc.] in a way that makes fewer assumptions about the form of relationship. In other words, we are estimating the relationship as locally weighted fits between the response and predictor variables. The locally weighted fits minimize the variance of the residuals or predictor error term. The technique of locally weighted regression function provides a smooth curve, the estimates of which are determined only by the data itself. It makes no assumption about the form of the relationship between response and predictor variables and allows the form to be discovered by the data itself.

(3) y=g(x)+ε

where,

***y*** = Proportion of population hesitant to COVID-19 vaccines

***x*** = Female and Male years of schooling; Female and Male access to the Internet; Multi-Dimensional Deprivation Index respectively

ε = Error term.

## 3. Results

The results of regression models are provided in (Table 1). We estimated two separate models, linear and log, within the demand and supply framework of vaccine uptake, to probe whether vaccine hesitancy, access to the internet and multi-dimensional deprivations play any statistically significant role in determining COVID-19 vaccination uptake or coverage. Our results show first, vaccine hesitancy has a negative and statistically significant impact on COVID-19 vaccination coverage (*p* < 0.01, Model 1 and 2). The elasticity of vaccine hesitancy (percentage change in the proportion of the population fully vaccinated due to a percentage change in vaccine hesitancy) is quite high. A percentage increase in vaccine hesitancy can lead to a decline in vaccination coverage by 30 percent (Model 2). Second, we have used deprivation faced by people (MPI) as a separate regressor in the models. As expected, we found a negative and statistically significant association between the MPI and the proportion of the population fully vaccinated in states (*p* < 0.05). A unit increase in MPI or proportion of people living in acute poverty leads to a mean decline in vaccination coverage by 50 percent. It implies that an increase in socioeconomic deprivation or the proportion of people living in multi-dimensional poverty negatively impacts health outcomes, including vaccination coverage. People living in acute poverty tend to give the least priority to their health, especially preventive care such as vaccination due to economic compulsions. They cannot afford to miss work and wage days for purposes like vaccination [16]. All these factors contribute to lower vaccine uptake among poor households. Third, access to the internet is statistically significant but negatively associated with female access and positively associated with male access. It implies that as male access to the internet increases, vaccination coverage also increases. The coefficient for male access to the internet is positive (0.6, *p* < 0.01). A percentage increase in the proportion of males with access to the internet causes a 60 percent increase in vaccination coverage. 

However, as female access to the internet increases, vaccination coverage decreases. The coefficient for female access to the internet is negative (0.53, *p* < 0.01). A percentage increase in the proportion of females with access to the internet causes a 50 percent decline in vaccination coverage, similarly. 

### 3.1. Determinants of COVID-19 Vaccine Coverage in India

#### Scatter Plots with Bivariate Regression Fits

**Figure 1:**
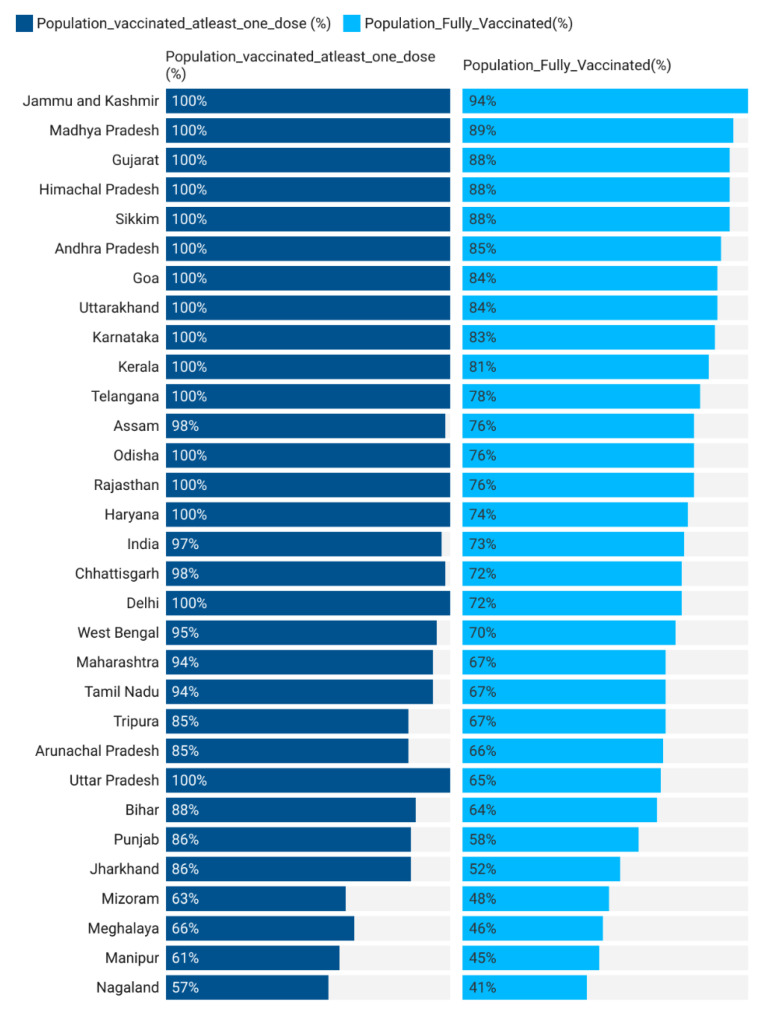
Progress of COVID-19 vaccination in India.

Vaccine Hesitancy: India’s COVID-19 vaccination rate has averaged 14 million doses per month since the inception of the vaccination program. A few states have performed consistently well by vaccinating 90–100 percent of their eligible population, while many states are yet to achieve the level of 60–70 percent coverage (Figure 1). Our model suggests that vaccine hesitancy is one of the potential reasons for low coverage in lag states. As shown, a percentage increase in vaccine hesitancy can lead to a decline in vaccination coverage by 30 percent.

**Figure 2:**
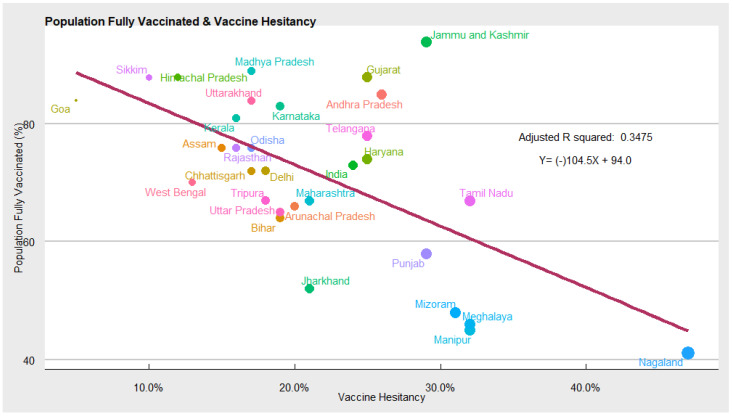
Scatter plot depicting COVID-19 vaccine hesitancy and vaccination coverage.

To get a complete picture of COVID-19 vaccine hesitancy and understand the trends of hesitancy across states, we analysed the findings of the CSS survey between January and December 2021. We found that COVID-19 vaccine acceptance rates in India vary between 53% and 95% among different states (Figure 2). Using the same CSS dataset, another study in India estimated that at the all-India level, about 45% of those surveyed would definitely choose to accept COVID-19 vaccines. However, a significant proportion of the individuals (29%) showed hesitancy in taking up the vaccine. Among the hesitant individuals, the top three reasons behind their hesitancy were the following: waiting and watching to see if the vaccine is safe; concern regarding side effects; other people needing more than me [17]. In one of the recent studies, the authors used the CSS data to report an increased risk of COVID-19 related outcomes among respondents living with a child attending school in person [18].

In another study using the CSS survey, the authors demonstrated the gender disparities in major health-related and non-health-related indicators at the regional and global levels. Using the CSS survey, this study highlighted that women reported significantly higher vaccine hesitancy (25.6%) compared with men (22.3%). The gender gap decreased over time, and by September 2021, it had largely closed at the global level [19]. Another study, using the CSS survey, in the USA, explored the factors associated with COVID-19 vaccine hesitancy and reported that gender is closely associated with vaccine hesitancy, with women being more hesitant. younger age and non-Asian were related to greater hesitancy [20].

Our analysis reveals that the Indian states that have struggled to achieve a higher strike rate of COVID-19 vaccination have also suffered from high COVID-19 vaccine hesitancy (Figure 2). For instance, states such as Tamil Nadu, Punjab, Nagaland, Meghalaya, Mizoram, Manipur, Jharkhand, etc. have high vaccine hesitancy and relatively low vaccination coverage. While states such as Himachal Pradesh, Sikkim, Goa, Madhya Pradesh, Kerala, Uttarakhand, etc. have low vaccine hesitancy and high coverage. To explore the causation between vaccine hesitancy, as measured by the CSS survey, and the COVID-19 vaccination coverage (% of the population fully vaccinated), we used a linear regression model. Controlling for other covariates, we found a negative and statistically significant relationship between the two, implying that COVID-19 vaccine hesitancy affects vaccination coverage negatively. Hence, to increase vaccination coverage in the lag states, measures to counter vaccine hesitancy should be accorded high priority.

**Figure 3:**
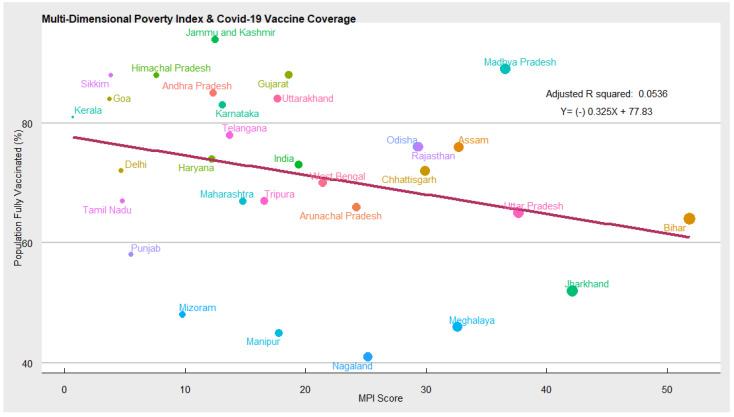
Scatter plot depicting MPI and vaccination coverage.

Socio-economic factors (Multi-Dimensional Deprivation Index): In Figure 3 we have used the multi-dimensional poverty index, computed by NITI AAYOG, as a proxy variable for socioeconomic conditions [15]. The MPI, originally developed by the Oxford Poverty and Human Development Initiative and adopted by the United Nation Development Program (UNDP), is an international measure of acute multidimensional poverty. It goes beyond the traditional monetary poverty measures and captures deprivations across the following three broad areas: health, education, and standard of living, which an individual faces. If an individual is deprived of more than 1/3rd of the total 12 indicators, he is considered multi-dimensionally poor [21]. In other words, MPI measures the extent and depth of poverty in India. The index also provided a comprehensive picture of people living in poverty across the states of India, permitting comparison. As expected, we found a negative association between the multi-dimensional poverty index and COVID-19 vaccination coverage. Our regression estimates reveal that socioeconomic deprivation plays a significant role in determining COVID-19 vaccination coverage. We find that MPI has a negative impact on COVID-19 vaccination coverage. This means as the proportion of the population living in multidimensional poverty increases, the COVID-19 vaccination coverage, measured by the percentage of the population fully vaccinated, decreases. The elasticity of MPI is quite high.

**Figure 4:**
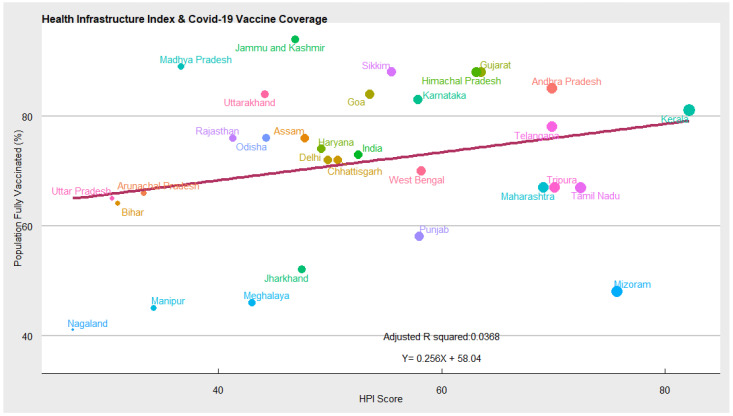
Scatter plot depicting state of health infrastructure and vaccination coverage.

Supply-Side Factors (Health Infrastructure): In Figure 4 the ‘state of health infrastructure’ and COVID-19 vaccination coverage among Indian states are depicted. The ‘state of the health infrastructure is measured by NITI AAYOG using the following three domains: (a) health outcomes (child mortality, immunisation coverage, institutional deliveries, disease prevalence, etc.); (b) health governance (full-time medical staff, financial resources, healthcare workers); (c) health system/service delivery (shortfall of healthcare workers, hiring of regular staff, the proportion of the following public health facilities: primary health centers (PHCs), community health centers (CHCs), district hospitals, total health expenditure, etc.). States with better health infrastructure scored higher in the health performance index.

Intuitively, states with better healthcare infrastructure are equipped to handle the COVID-19 crisis effectively and inoculate individuals at a faster rate. Controlling for other covariates, we found a positive correlation between health infrastructure and COVID-19 vaccination coverage. This means the COVID-19 vaccination rate will increase as states improve their health infrastructure. For instance, states such as Kerala, Andhra Pradesh, Karnataka, Himachal Pradesh, Sikkim, Gujrat, Goa, etc. have better health infrastructure and relatively high vaccination coverage. While states such as Nagaland, Manipur, Meghalaya, Jharkhand, Bihar, Arunachal Pradesh, Uttar Pradesh, etc. have poor health infrastructure and low vaccination coverage.

**Figure 5:**
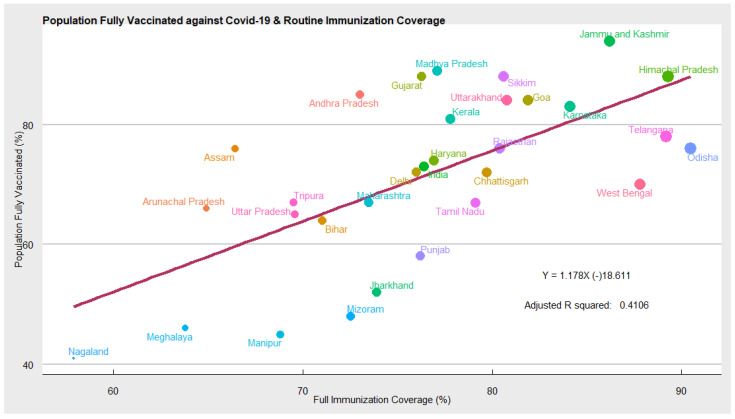
Scatter plot depicting routine immunisation and COVID-19 vaccination coverage.

Routine Immunisation Coverage: In Figure 5 we explored whether the acceptance of routine child immunisation, measured as the proportion of the child population fully immunized, has a role in determining the likelihood of accepting adult vaccine-like COVID-19 or not. Using the regression model, we found that acceptance of childhood vaccination does have a positive impact on the likelihood of accepting COVID-19 vaccines. The scatter plot and regression line suggests a positive relationship between the two variables. Our results show that in states such as Himachal Pradesh, Jammu, Kashmir, Karnataka, Goa, Sikkim, Telangana, etc., child immunisation coverage and high COVID-19 vaccine coverage. While, Jharkhand, Bihar, Meghalaya, Meghalaya, Nagaland, Mizoram, Punjab, Manipur, etc. have relatively low coverage of both routine and COVID-19 vaccination.

**Figure 6:**
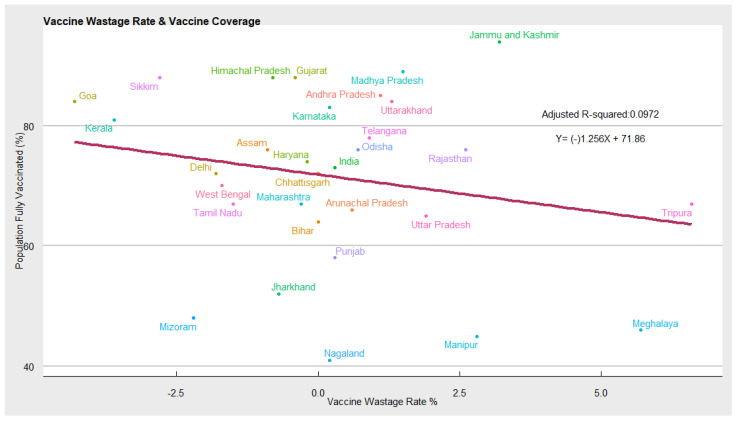
Scatter plot depicting vaccine wastage and COVID-19 vaccination coverage.

Vaccine Wastage: In Figure 6 the vaccine wastage, measured as the difference between total COVID-19 vaccinations supplied and utilised by the state, is an important indicator of COVID-19 vaccine demand and the state’s health infrastructure (capacity to utilise the vaccines). States with higher demand and better healthcare infrastructure will report fewer vaccine wastages compared with states with low demand and weak health infrastructure. Our estimates show a negative correlation between the COVID-19 vaccination coverage and wastage. This means states with high vaccination coverage report lower vaccine wastage. While states with low vaccination coverage report high wastages.

### 3.2. Determinant of Vaccine Hesitancy in India

#### Scatter Plots with Loess Regression Fits

Next, we explored the factors that may have an individual impact on COVID-19 vaccine hesitancy. According to WHO, vaccine hesitancy is context-specific and varies with time, space, and disease. The WHO Strategic Advisory Group of Experts (SAGE) defines vaccine hesitancy as “delay in acceptance or refusal of vaccination despite the availability of vaccination services”. Interestingly, vaccine hesitancy exists to differing degrees in different countries, depending on the extent of the health and socioeconomic situation in each country. Factors such as religion, gender, political ideology and trust in medical and scientific institutions are associated with vaccine hesitancy, both in general and regarding COVID-19 vaccines specifically [22,23]. Socioeconomic and racial inequities pertaining to health disparities during the pandemic persist. Minorities, individuals in lower-income quintiles, and less educated individuals (college degree or lower) are disproportionately more susceptible to COVID-19 and have considerably lower vaccine acceptance. In the USA, individuals without a college degree are 42% less likely to get vaccinated. Individuals with lower incomes living in rural areas are also less likely to get vaccinated [22].

**Figure 7:**
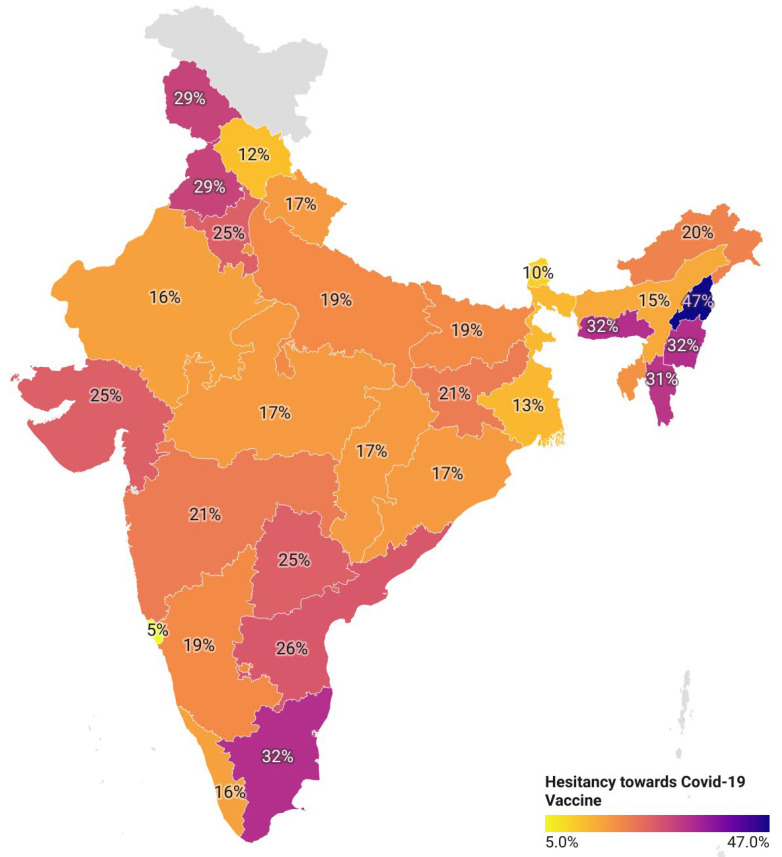
Trends of vaccine hesitancy in India.

In India, safety concerns, suspicions towards new vaccines, the pace at which COVID-19 vaccines are developed and granted approval for human use, limited data about the safety of vaccines, rumours, and misinformation on social media influence the public trust in COVID-19 vaccines and may cause anxiety among people. Recently, people have been debating the credibility of vaccines against emerging variants such as Omicron. Another dominant reason behind vaccine hesitancy is fear of its side effects. A section (across religions) of the population believed vaccines to be against their religion-cultural realm. As a result, there is an urgent need to understand the determinants of vaccine hesitancy and the factors that influence people’s willingness to accept or reject COVID-19 vaccines. In Figure 7 the geographic map of India shows varying degrees of vaccine hesitancy and large disparities between states, with the highest rate of vaccine hesitancy in Nagaland (47%) and the lowest in Goa (5%).

**Figure 8:**
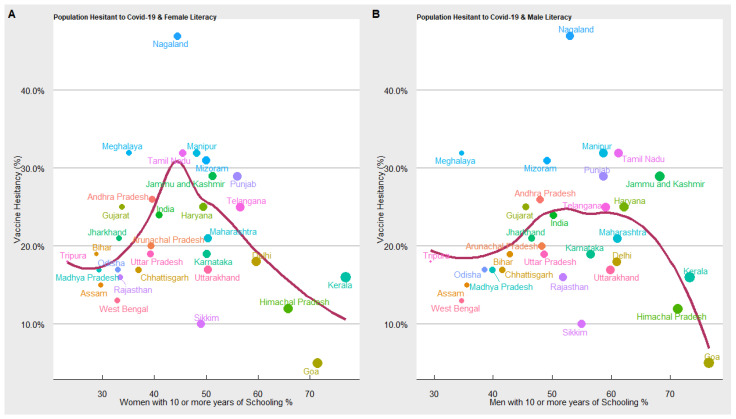
Results of vaccine hesitancy and literacy.

Literacy—10 or more years of schooling: In Figure 8 we used the “loess regression model” to estimate the relationship between COVID-19 vaccine hesitancy and years of schooling (male and female). We have estimated two separate models to explore whether years of schooling impact people’s awareness and willingness to accept or reject the COVID-19 vaccine. We find an inverted-U-shaped relation between female and male years of schooling and COVID-19 vaccine hesitancy. In the initial stages, when the proportion of the population with 10 or more years of schooling is low, COVID-19 vaccine hesitancy increases, but beyond a threshold level; when a significant proportion of the population is literate, the trend reverses, such that at a higher level of literacy, the COVID-19 vaccine hesitancy declines. What also emerges from our results is that developed states with a higher proportion of literate males and females tend to have a lower rate of COVID-19 vaccine hesitancy.

**Figure 9:**
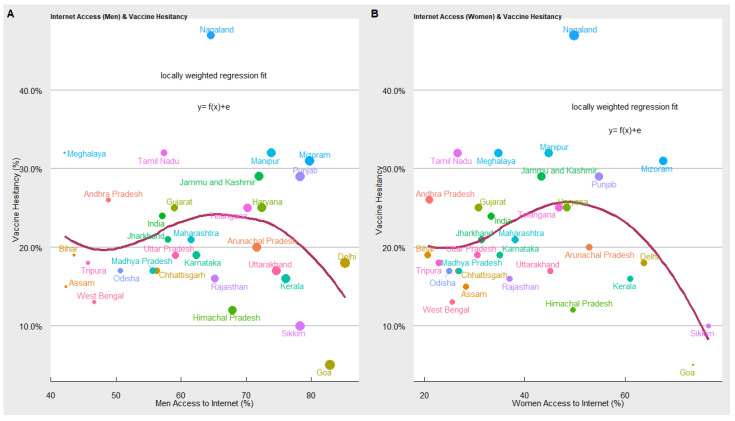
Results of vaccine hesitancy and access to the internet.

Access to the Internet: In Figure 9 we explored the relationship between COVID-19 vaccine hesitancy and access to the internet in the states. In the below figure, the *x*-axis represents the proportion of the population (male and female) with Internet access and the *y*-axis represents the proportion of the population hesitant toward the COVID-19 vaccine. We used the loess regression model to estimate the relationship between COVID-19 vaccine hesitancy and male and female access to the internet. The scatter plot and the loess regression function suggest an inverted-U relationship between access to the internet and vaccine hesitancy. When access to the internet is low, vaccine hesitancy is high, but at some level of internet access (different for males and females), the trend reverses, and higher access to the internet leads to a decline in COVID-19 vaccine hesitancy. Our results show that states such as Delhi, Kerala, Sikkim, Goa, Himachal Pradesh, Haryana, Karnataka, etc., have high internet penetration and low vaccine hesitancy. While Jharkhand, Bihar, Meghalaya, Tamil Nadu, Andhra Pradesh, etc., have relatively low access to the internet and high vaccine hesitancy. Some states have high vaccine hesitancy despite having higher access to the internet. It implies access to the internet can increase or decrease the likelihood of getting vaccinated. Available literature also suggests that the availability of digital tools such as the internet and smartphones helps in enhancing awareness and access to vaccines. However, it also presents several challenges, mainly rumours, misinformation, anti-vax sentiments, fake news and incomplete information regarding vaccines [7].

**Figure 10:**
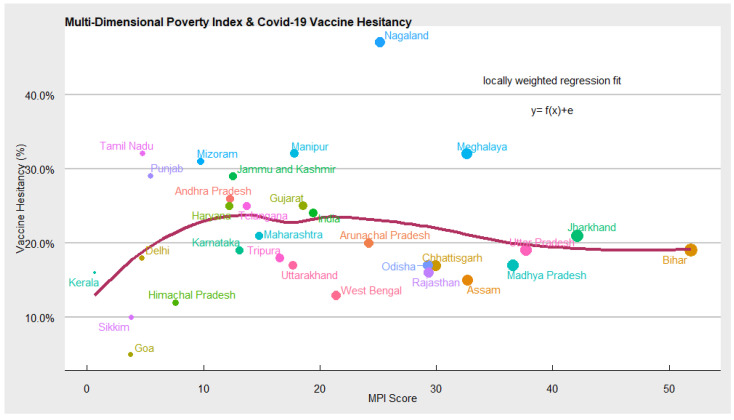
Results of vaccine hesitancy and MPI.

Multi-Dimensional Deprivation Index: In Figure 10 using loess regression, we further explored the relationship between vaccine hesitancy, as indicated in the CSS survey, and the multi-dimensional poverty index of Niti Aayog, measuring the acute multi-dimensional poverty. The scatter plot and the loess regression function suggest no statistically significant association between vaccine hesitancy and MPI. At a lower level of MPI, i.e., a lower proportion of the population living in acute poverty, COVID-19 vaccine hesitancy is also low. However, as the ‘MPI poor’ increases, vaccine hesitancy stagnates, implying no impact. The states such as UP, Bihar, Madhya Pradesh, Jharkhand, etc. have the highest number of people living in multidimensional poverty, but the level of vaccine hesitancy in these states is similar to states such as Delhi, Haryana, Karnataka, Telangana, Gujarat, etc. with few people living in acute poverty. This can be due to the successful efforts of the Union and State governments in disseminating information on the safety and efficacy of COVID-19 vaccines.

## 4. Discussion

In the present analysis, we first explored the factors determining COVID-19 vaccine coverage in India and estimated the impact of social and economic variables on COVID-19 vaccination coverage. We further delved into the factors that may have an individual impact on COVID-19 vaccine hesitancy using a separate regression model. We found that in general, states which have struggled to achieve a higher strike rate of vaccination coverage have also suffered from high COVID-19 vaccine hesitancy. Our result shows that a percentage increase in vaccine hesitancy can lead to a decline in vaccination coverage by 30 percent. Thus, COVID-19 vaccine hesitancy is one of the potential reasons for low vaccination coverage in some lag states. It implies that policies must focus on mitigating COVID-19 vaccine hesitancy to achieve the target of universal immunisation against COVID-19. Next, we probed whether state-level socioeconomic disparities, as measured by MPI, have any association with COVID-19 vaccination coverage. The extent of deprivation (MPI) is high in states such as Uttar Pradesh, Bihar, Jharkhand, Madhya Pradesh, Chhattisgarh, Rajasthan, Assam, Meghalaya, Nagaland, Odisha, etc. Conversely, the extent of deprivation is comparatively lower in states such as Kerala, Delhi, Goa, Sikkim, Himachal Pradesh, Karnataka, Andhra Pradesh, Haryana, etc. [15]. Importantly, states which have been investing considerably in the health and education sectors over the past few decades have not only been performing consistently well in socioeconomic development, nutrition, child mortality, antenatal care, schooling, sanitation, water, electricity, housing, economic assets, and medical infrastructure. However, maintaining a robust healthcare infrastructure enables them to manage emergency health crises such as COVID-19 effectively. As expected, we found a negative correlation between socioeconomic conditions as captured by the multi-dimensional poverty index and COVID-19 vaccination coverage. Reduction in multi-dimensional poverty not only improves the standard of living of the people but changes their behaviour towards health, education, diseases, etc. It has been widely seen that as the socioeconomic condition improves, people’s spending (time and money) on healthcare and education increases. Furthermore, due to rising awareness about health, the overall health outcomes, including health infrastructure, in the states improve, which has a positive implication on the management of emergencies such as COVID-19 and enhancing vaccination coverage. We additionally demonstrated that male and female access to digital technologies such as access to the smart phones and the internet is statistically significant and is associated differently with COVID-19 vaccination coverage. For instance, we found that, as male access to the internet increases, vaccination coverage also increases. This is attributed to (a) the widening digital divide between males and females and India’s reliance on using digital tools (COWIN, AAROGYA SETU) to allocate and register for COVID-19 vaccines. With males having greater digital access than females it provided them with an added advantage in timely registration, locating vaccination centres, accessing vaccine certificates, etc. (b) Gender-based disparity in utilization of the COVID-19 vaccines, of the total 1.7 billion doses administered in India, males have received 870 million or 51%, while females have received 820 million or 48% [24]. The gender disparity in COVID-19 vaccination implies that a greater number of males are getting vaccinated than female in absolute terms and this difference is getting reflected in the results (Since more male are getting vaccinated, any factor that increases the male access to COVID-19 vaccination have a larger impact on absolute coverage). Conversely, female access to the internet is statistically significant and negatively associated with vaccination coverage. This can be attributed to (a) higher vaccine hesitancy among the female population. Multiple surveys conducted by organisations such as IDFC Institute, Mumbai [25], NCAER [17], etc., highlighted that females are likely to be more hesitant about COVID-19 vaccines due to the spread of rumours on social media [26]. Females are more prone to believing in misinformation and rumours regarding vaccines’ side effects, infertility, and menstruation. (b) Females are less likely to utilise healthcare services and household resources on health. Structural problems in access to healthcare and gendered-biased household resource allocation to female health have resulted in neglect of female health in India. There are strong perceptions across households that since men go out to work, their health must be protected and prioritized over women who mostly engage in domestic work.

Our results resonate with previous research conducted in India and abroad. For instance, a population-based longitudinal survey, conducted on 3000 participants across four Indian states (June 2021) to map COVID-19 vaccine acceptance, hesitance, and resistance, reported only one in two Indians (58%) were definitely getting vaccinated. The survey divided vaccine hesitancy into low (28% who will probably get vaccinated) and high (7% who will not get vaccinated) levels. The survey further reported that covariates such as age, gender, geography, education level, and economic status are associated with vaccine hesitancy. As per the survey, females were more vaccine-hesitant, and respondents holding a graduate degree and above were more in favor of getting vaccinated compared with respondents who had less than 10 years of schooling. Respondents living in high-income households were more likely to be vaccinated compared to low-income households [27]. A cross-country analysis assessing the association between the socio-economic factors: age, gender, education, and vaccine acceptance in high-COVID-19 burden countries reveals that women in countries such as France, Germany, and Sweden are more likely to accept a vaccine than men. Older adults (greater than 50) in Canada, Poland, France, Germany, and the UK are more eager to get the vaccines than younger adults. Similarly, educated individuals in India, Ecuador, the USA, and Germany are likely to accept the vaccine. However, higher education levels are negatively associated with vaccines in Canada, Spain, and the UK [28].

We further probed the determinants that could be associated with the unwillingness to get vaccinated against COVID-19. We expected higher literacy to be associated with better health outcomes as people have knowledge and awareness regarding diseases, morbidity factors, and general health status, including vaccination. We found that literacy is an inverted U function of vaccine hesitancy. This means that states with a low level of literacy tend to have a high level of vaccine hesitancy. Interestingly, our results show that initially, as the literacy level increases, vaccine hesitancy also increases. However, once a larger proportion of the population attains literacy, the trend reverses and vaccine hesitancy declines. The result has important policy implications; vaccination awareness campaigns have a positive impact once a certain threshold of the population is literate enough to understand the importance of health programs such as vaccination. This means that measures to mitigate vaccine hesitancy should be local and designed keeping in view the level of general literacy in the target states. We further demonstrated that access to digital technologies such as the internet is an important determinant of willingness to accept or reject COVID-19 vaccines. Our results show that access to digital technologies has both negative and positive impacts. Having access to the Internet aids in timely registration for vaccine slots, locating nearby vaccination centres, accessing digital vaccine certificates, etc. However, it can also reduce the chances of getting vaccinated as people can get influenced by the rampant misinformation and rumours’ on COVID-19 vaccines. Our results are similar to Machingaidze and Wiysonge, 2021, who argued that the wide availability of digital tools such as the internet and smartphones helped in enhancing access to the internet and social media in LMICs. Although this is a great tool for self-education, awareness, access to vaccines, etc., which are key components of vaccination decision-making, it also presents several challenges, mainly rumours, misinformation, anti-vax sentiments, fake news, and incomplete information regarding vaccines.

The empirical results from our study can be used by policymakers to design and improve COVID-19 mitigation strategies. A significant proportion of the population remains vaccine-hesitant in many Indian states. Vaccine hesitancy is significantly influenced by the number mean years of schooling and access to digital technologies such as the internet. The widespread misinformation on the internet is fueling vaccine hesitancy in many states and, thus, strategies that could potentially target the spread of misinformation need to be adopted. In areas with a low level of literacy, COVID-19 vaccine awareness campaigns may be started. In the medium run, the uptake of healthcare services, including vaccination, is associated with investments made in healthcare infrastructure and socioeconomic indicators. States that have been investing considerably in the health and education sectors over the past few decades have been maintaining a robust healthcare infrastructure, enabling them to manage emergency health crises such as COVID-19 effectively.

The COVID-19 crisis has overwhelmed the healthcare system in the country and posed serious health infrastructure challenges for the government. The belief that urban healthcare infrastructure is equipped to handle emergencies stands busted. This is imperative in rural areas where the healthcare infrastructure is more underdeveloped. The subsequent COVID-19 waves have taught us that we need to intensify our healthcare infrastructure both in urban and rural areas. The healthcare infrastructure plays a crucial role in mitigating healthcare emergencies such as COVID-19. States with better healthcare infrastructure are equipped to handle the COVID-19 crisis effectively and inoculate individuals at a faster rate. Controlling for other covariates, we found a positive correlation between health infrastructure and COVID-19 vaccination coverage. This means the COVID-19 vaccination rate will increase as states improve their health infrastructure.

## 5. Conclusions

The paper suggests strong linkages between COVID-19 vaccination coverage and factors such as vaccine hesitancy, socioeconomic condition, health infrastructure, literacy, and access to the internet. The major findings of the paper suggest that states with low vaccine hesitancy, less acute poverty, better health infrastructure, literacy, etc., achieved higher COVID-19 vaccination coverage. While states with high vaccine hesitancy, a higher proportion of people living in acute poverty, poor health infrastructure, etc., had low COVID-19 vaccination coverage.

## Figures and Tables

**Table 1:** Multiple linear regression results.

Independent Variables	Dependent Variable:	
	Population Fully Vaccinated (Model 1)	Log of Population Fully Vaccinated (Model 2)
Vaccine Hesitancy	−111.764 *** (′ 24.55)	
Multi-dimensional Deprivation Index	−0.508 ** (′ 0.2)	
Female Access to the Internet	−0.594 ** (′ 0.273)	
Male Access to the Internet	0.423 (′ 0.339)	
Vaccine Wastage Rate	0.054 (′ 0.996)	
Log of Vaccine Hesitancy		−0.303 *** (′ 0.074)
Log of Multi-dimensional Deprivation Index		−0.075 * (′ 0.043)
Log of Female Access to the Internet		−0.534 *** (′ 0.175)
Log of Male Access to the Internet		0.689 ** (′ 0.313)
Constant	103.105 *** (′ 17.1)	3.064 *** (′ 0.934)
Observations	31	31
R2	0.556	0.518
Adjusted R2	0.467	0.444
Residual Std. Error	10.369 (df = 25)	0.163 (df = 26)
F Statistic	6.250 *** (df = 5; 25)	6.994 *** (df = 4; 26)

Note: ′ indicates level of significance; * *p* < 0.1; ** *p* < 0.05; *** *p* < 0.01 (df indicates degree of freedom).

## Data Availability

The data are available in public domain and can be downloaded after putting forward a request.
